# The Role of Thiamine Deficiency in Alcoholic Brain Disease

**Published:** 2003

**Authors:** Peter R. Martin, Charles K. Singleton, Susanne Hiller-Sturmhöfel

**Affiliations:** Peter R. Martin, M.D., is professor of psychiatry and pharmacology at Vanderbilt University School of Medicine and director of the Vanderbilt Addiction Center, Nashville, Tennessee. Charles K. Singleton, Ph.D., is professor and chair in the Department of Biological Science, Vanderbilt University, Nashville, Tennessee. Susanne Hiller-Sturmhöfel, Ph.D., is a science editor for *Alcohol Research & Health*

**Keywords:** thiamine deficiency, alcoholic brain syndrome, chronic AODE (alcohol and other drug effects), Wernicke’s encephalopathy, Wernicke-Korsakoff psychosis, alcoholic cerebellar degeneration, AODR (alcohol and other drug related) structural brain damage, malnutrition, disease susceptibility, survey of research

## Abstract

A deficiency in the essential nutrient thiamine resulting from chronic alcohol consumption is one factor underlying alcohol-induced brain damage. Thiamine is a helper molecule (i.e., a cofactor) required by three enzymes involved in two pathways of carbohydrate metabolism. Because intermediate products of these pathways are needed for the generation of other essential molecules in the cells (e.g., building blocks of proteins and DNA as well as brain chemicals), a reduction in thiamine can interfere with numerous cellular functions, leading to serious brain disorders, including Wernicke-Korsakoff syndrome, which is found predominantly in alcoholics. Chronic alcohol consumption can result in thiamine deficiency by causing inadequate nutritional thiamine intake, decreased absorption of thiamine from the gastrointestinal tract, and impaired thiamine utilization in the cells. People differ in their susceptibility to thiamine deficiency, however, and different brain regions also may be more or less sensitive to this condition.

Alcohol consumption can damage the brain through numerous mechanisms, many of which are discussed in the articles in this issue of *Alcohol Research & Health*. One of these mechanisms involves the reduced availability of an essential nutrient, thiamine, to the brain as a consequence of chronic alcohol consumption. This article describes the normal role of thiamine in brain functioning as well as the pathological consequences that result from thiamine deficiency. Specific actions of thiamine on a cellular level then are reviewed, followed by a discussion of how alcohol affects the body’s processing and availability of thiamine as well as thiamine utilization by the cells. Finally, the article explores the hypothesis that people may differ in their sensitivity to thiamine deficiency and that different brain regions may be more or less sensitive to a deficiency in this important nutrient. Thiamine deficiency is particularly important because it can exacerbate many of the other processes by which alcohol induces brain injury, as described in other articles in this issue of *Alcohol Research & Health.*

## What is Thiamine and What are the Consequences of Thiamine Deficiency?

Thiamine, also known as vitamin B_1_, is an essential nutrient required by all tissues, including the brain. The human body itself cannot produce thiamine but must ingest it with the diet. Thiamine-rich foods include meat (e.g., pork) and poultry; whole grain cereals (e.g., brown rice and bran); nuts; and dried beans, peas, and soybeans. In addition, many foods in the United States commonly are fortified with thiamine, including breads and cereals. Humans require a minimum of 0.33 milligrams (mg) thiamine for every 1,000 kilocalories (kcal) of energy they consume—in other words, people who consume a regular 2,000-kcal diet per day should ingest a minimum of 0.66 mg thiamine daily (Hoyumpa 1980). To provide a safety margin, a daily intake of 1.1 mg thiamine is currently recommended for adult women and 1.2 mg for adult men.^1^ Studies have found that most healthy people typically consume 0.4 to 2.0 mg thiamine daily (Woodhill and Nobile 1972).

In the body, particularly high concentrations of thiamine are found in skeletal muscles and in the heart, liver, kidney, and brain (Singleton and Martin 2001). In the tissues, thiamine is required for the assembly and proper functioning of several enzymes that are important for the breakdown, or metabolism, of sugar molecules into other types of molecules (i.e., in carbohydrate catabolism). Proper functioning of these thiamine-using enzymes is required for numerous critical biochemical reactions in the body, including the synthesis of certain brain chemicals (i.e., neurotransmitters); production of the molecules making up the cells’ genetic material (i.e., nucleic acids); and production of fatty acids, steroids, and certain complex sugar molecules. In addition, inadequate functioning of the thiamine-using enzymes can interfere with the body’s defense against the damage (i.e., oxidative stress) caused by harmful, highly reactive oxygen molecules called free radicals. (For more information, see the section “Thiamine’s Actions in the Cell.”)

Because thiamine and the thiamine-using enzymes are present in all cells of the body, it would be plausible that inadequate thiamine affects all organ systems; however, the cells of the nervous system and heart seem particularly sensitive to the effects of thiamine deficiency. Therefore, the resulting impairment in the functioning of the thiamine-using enzymes primarily affects the cardiovascular and nervous systems. The classical manifestations of thiamine deficiency–related heart disease include increased blood flow through the vessels in the body, heart failure, and sodium and water retention in the blood. In the brain, thiamine is required both by the nerve cells (i.e., neurons) and by other supporting cells in the nervous system (i.e., glia cells). Thiamine deficiency is the established cause of an alcohol-linked neurological disorder known as Wernicke-Korsakoff syndrome (WKS), but it also contributes significantly to other forms of alcohol-induced brain injury, such as various degrees of cognitive impairment, including the most severe, alcohol-induced persisting dementia (i.e., “alcoholic dementia”). These disorders are discussed in the following sections.

### Wernicke’s Encephalopathy and Korsakoff ’s Psychosis

WKS typically consists of two components, a short-lived and severe condition called Wernicke’s encephalopathy (WE) and a long-lasting and debilitating condition known as Korsakoff ’s psychosis. WE is an acute life-threatening neurologic disorder caused by thiamine deficiency. In affluent countries, where people normally receive adequate thiamine from their diets, thiamine deficiency is most commonly caused by alcoholism (Singleton and Martin 2001); accordingly, in these countries WE is primarily found in alcoholics (Ragan et al. 1999). The symptoms of WE include mental confusion, paralysis of the nerves that move the eyes (i.e., oculomotor disturbances), and an impaired ability to coordinate movements, particularly of the lower extremities (i.e., ataxia). For example, patients with WE may be too confused to find their way out of a room or may not even be able to walk. Many WE patients, however, do not exhibit all three of these signs and symptoms, and clinicians working with alcoholics must be aware that WE may be present even if the patient presents with only one or two of them. In fact, neuropathological studies after death indicate that many cases of thiamine deficiency–related encephalopathy may not be diagnosed in life because not all the “classic” signs and symptoms are present or recognized.

Approximately 80 to 90 percent of alcoholics with WE develop Korsakoff ’s psychosis, a chronic neuropsychiatric syndrome characterized by behavioral abnormalities and memory impairments (Victor et al. 1989). Although these patients have problems remembering old information (i.e., retrograde amnesia), it is the disturbance in acquisition of new information (i.e., anterograde amnesia) that is most striking. For example, these patients can engage in a detailed discussion of events in their lives but cannot remember ever having had that conversation an hour later. Because of these characteristic memory deficits, Korsakoff ’s psychosis also is called alcohol amnestic disorder. It is still somewhat controversial, however, whether Korsakoff ’s psychosis always is preceded by WE or whether it develops in fits and starts, without an overt episode of WE.

The role of thiamine in the development of WKS is supported by findings that giving this nutrient to patients with WKS reverses many of the acute symptoms of the disease, although in some people certain chronic neuropsychiatric consequences of previous thiamine deficiency may persist even with appropriate treatment (see Singleton and Martin 2001). In the most severe cases, these persistent symptoms meet the criteria of full-blown Korsakoff ’s psychosis. Other people may exhibit more subtle neurological signs and symptoms, such as abnormalities in a brain region called the cerebellum (as described in the following section) and an inflammation or degeneration of peripheral nerves (i.e., neuropathy) as well as changes in behavior and problems with learning, memory, and decisionmaking.

**Figure 1:**
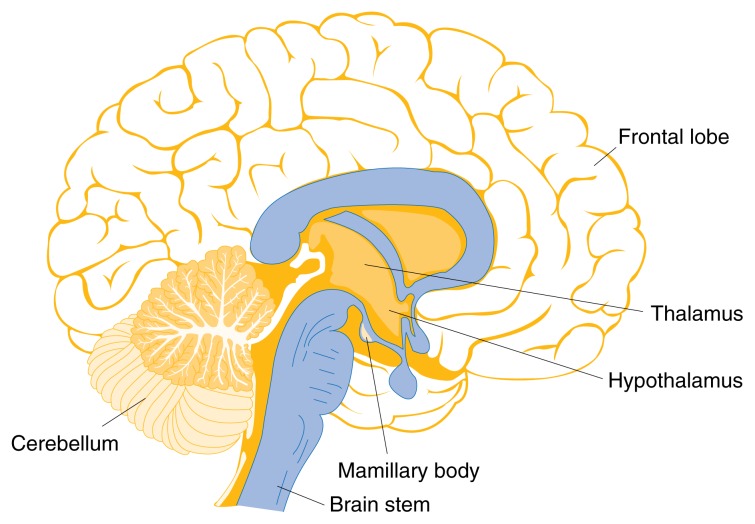
Brain regions affected by thiamine deficiency include the cerebellum, mamillary bodies, thalamus, hypothalamus, and brain stem.

In affluent countries such as the United States, where other forms of malnutrition are uncommon, thiamine deficiency and the resulting WKS occur most commonly among alcoholics. To date there are only a few estimates of how common WKS is among alcoholics. In autopsy studies, brain abnormalities characteristic of WKS were present in approximately 13 percent of alcoholics (Harper et al. 1988). These abnormalities include lesions in brain areas called the mamillary bodies, thalamus, hypothalamus, brain stem, and cerebellum (see figure 1). Other studies have found that only about 20 percent of alcoholics in whom the presence of WKS was confirmed at autopsy had been diagnosed with the disorder before death (Harper 1998). Thus, the clinical presentation is not always easily recognized by physicians; often examination of the brain at autopsy is required for definitive diagnosis.

Although WKS in developed countries occurs most commonly among alcoholics, other groups of patients are also at risk of developing the disease. For example, all people who are malnourished (e.g., because they are HIV infected or are undergoing cancer chemotherapy) or who have a metabolic disease leading to impaired thiamine absorption (i.e., uptake) or utilization can develop thiamine deficiency. Patients with severe kidney disease who are undergoing regular dialysis are also prone to encephalopathy, and a substantial portion of them have been found to suffer from thiamine deficiency (Hung et al. 2001). Finally, patients who receive intravenous infusions of carbohydrates (e.g., the sugar dextrose) may experience episodes of thiamine deficiency, particularly if they are already at risk of receiving inadequate levels of this nutrient because they are alcoholics, as thiamine is used in the metabolism of those carbohydrates (see Ferguson et al. 1997).

### Cerebellar Degeneration

Considerably more common than WKS among alcoholics is a condition called cerebellar degeneration, which typically develops after 10 or more years of heavy drinking (Charness 1993). In autopsy studies, 40 percent or more of alcoholics showed signs of this condition (Torvik 1987), which is characterized by shrinkage (i.e., atrophy) of certain regions of the cerebellum. This brain area is involved primarily in muscle coordination. It also is increasingly recognized for its role in various aspects of cognitive and sensory functioning (Parks et al. 2003). Accordingly, cerebellar degeneration is associated with difficulties in movement coordination and involuntary eye movements, such as nystagmus. Cerebellar degeneration is found both in alcoholics with WKS and in those without it, but because WKS patients typically have a higher degree of cerebellar atrophy, it appears likely that thiamine deficiency also is the predominant cause of cerebellar degeneration.

The frequent occurrence of cerebellar degeneration in alcoholics is consistent with studies demonstrating that the cerebellum is particularly sensitive to the effects of thiamine deficiency. (For more information on these studies, see the section “Differential Sensitivity of Various Brain Regions.”) As a result of this particular susceptibility, the effects of thiamine deficiency would be expected to appear first in the cerebellum, manifesting as cerebellar degeneration and its associated symptoms. In a smaller number of patients, the consequences of insufficient thiamine then would progress to other brain regions and lead to more widespread brain dysfunction, including alcohol amnestic disorder or alcohol-induced persisting dementia.

## Thiamine’s Actions in the Cell

To understand the mechanisms through which thiamine deficiency, whether induced by alcoholism or other causes, leads to brain damage, one first must understand the normal role of thiamine in the cell. Investigations of this issue have focused on three enzymes that require thiamine as a cofactor. These enzymes are called transketolase, pyruvate dehydrogenase (PDH) and alpha-ketoglutarate dehydrogenase (α-KGDH); they all participate in the catabolism of sugar molecules (i.e., carbohydrates) in the body, as described in the following paragraphs. Each of these enzymes consists of several components that must be assembled to yield the functional enzyme, and the addition of thiamine is a critical step in this assembly process. As a result, thiamine deficiency causes suboptimal levels of functional enzymes in the cell, in addition to interfering with the activity of those enzymes.

**Figure 2:**
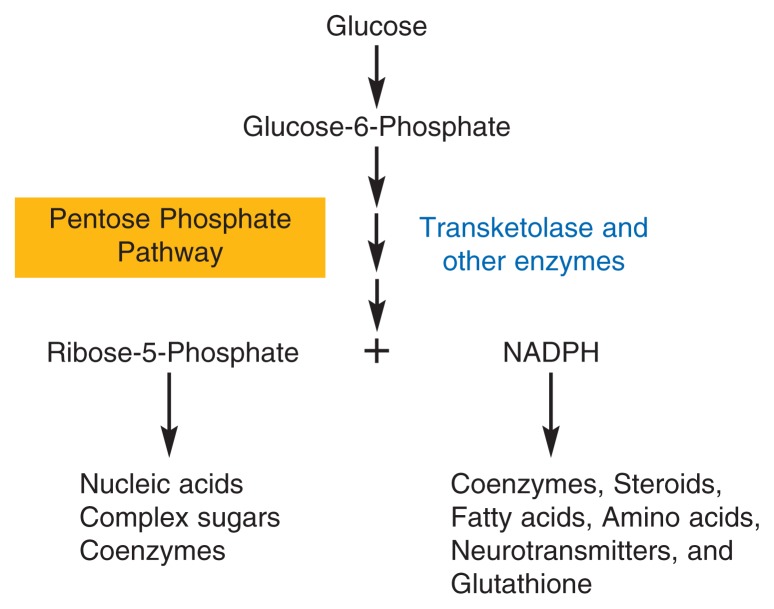
The thiamine-dependent enzyme transketolase is an important enzyme in the breakdown of glucose through a biochemical pathway called the pentose phosphate pathway. Glucose is first converted to a molecule called glucose-6-phosphate, which enters the pentose phosphate pathway where it is further modified by transketolase. During that reaction, two products are formed—the sugar ribose-5-phosphate and a molecule called reduced nicotinamide adenine dinucleotide phosphate (NADPH). Ribose-5-phosphate is needed for the synthesis of nucleic acids, complex sugar molecules, and other compounds called coenzymes that are essential for the functioning of various enzymes. NADPH provides hydrogen atoms for chemical reactions that result in the production of coenzymes, steroids, fatty acids, amino acids, and neurotransmitters. In addition, NADPH plays an important role in the synthesis of glutathione, a compound that is essential to the body’s defense against damage from oxidative stress. Reduced transketolase activity interferes with all these essential biochemical processes.

Transketolase is an important enzyme in a biochemical pathway called the pentose phosphate pathway. In this set of biochemical reactions, a molecule called glucose-6-phosphate, which is derived from the sugar glucose, is modified by transketolase, yielding two products—a sugar called ribose-5-phosphate and a molecule called reduced nicotinamide adenine dinucleotide phosphate (NADPH) (see figure 2). Both of these molecules are essential for the production of numerous other important molecules in the cell. Ribose-5-phosphate is needed for the synthesis of nucleic acids, complex sugar molecules, and other compounds. NADPH provides hydrogen atoms for chemical reactions that result in the production of steroids, fatty acids, amino acids, certain neurotransmitters, and other molecules. In addition, NADPH plays an important role in the synthesis of glutathione, a compound that is essential in the body’s defense against oxidative stress. To function properly, all cells require certain levels of NADPH and ribose-5-phosphate, and the biochemical reaction mediated by transketolase is crucial for maintaining the appropriate levels of both molecules.

**Figure 3:**
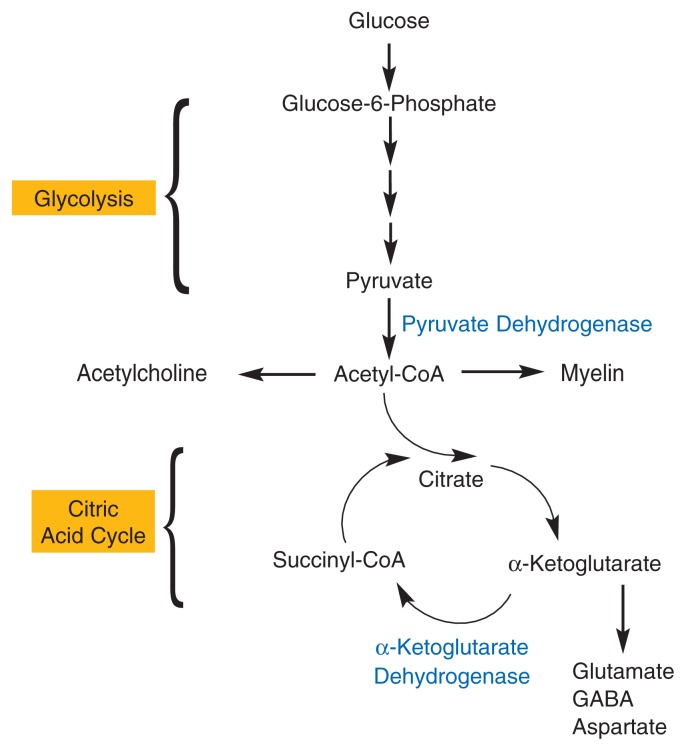
The thiamine-dependent enzymes pyruvate dehydrogenase (PDH) and α-ketoglutarate dehydrogenase (α–KGDH) participate in the metabolism of glucose through two biochemical reactions, glycolysis and the citric acid cycle. The main function of these two sets of reactions is to generate adenosine triphosphate (ATP), which provides energy for the cells. Reduced PDH and α–KGDH activity resulting from thiamine deficiency can lead to less ATP synthesis, which in turn can contribute to cell damage and even cell death. In addition, PDH is needed to produce the neurotransmitter acetylcholine and to generate myelin, a compound that forms a sheath around the extensions (i.e., axons) of many neurons, thereby ensuring proper neuronal functioning. The citric acid cycle and α–KGDH play a role in maintaining the levels of the neurotransmitters glutamate, gamma-aminobutyric acid (GABA), and aspartate, as well as in protein synthesis.

The other two enzymes requiring thiamine, PDH and α-KGDH, also participate in different steps of the breakdown and conversion of glucose-6-phosphate through two consecutive chains of biochemical reactions called glycolysis and the citric acid cycle (see figure 3). The main function of these pathways is the generation of a molecule called adenosine triphosphate (ATP), which provides energy for numerous cellular processes and reactions. Decreases in the activities of PDH and α-KGDH can result in reduced ATP synthesis, which in turn can contribute to cell damage and even cell death. In addition, proper functioning of PDH is essential for the production of the neurotransmitter acetylcholine as well as for the synthesis of a compound called myelin, which forms a sheath around the extensions (i.e., axons) of many neurons, thereby ensuring the ability of these neurons to conduct signals. The citric acid cycle and α-KGDH play a role in maintaining the levels of the neurotransmitters glutamate, gamma-aminobutyric acid (GABA), and aspartate, as well as in protein synthesis. Thus, the thiamine-using enzymes play numerous vital roles in the functioning of cells, and particularly of neurons.

When thiamine levels decrease, the activity levels of all three enzymes are reduced to some extent. The specific reductions depend both on the enzyme and on the cell type studied (Singleton and Martin 2001). Overall, transketolase activity may be the most sensitive measure of thiamine deficiency. Studies using rats found that transketolase activity may be reduced as much as 90 percent in the brain regions that are most sensitive to thiamine deficiency (Gibson et al. 1984). Substantial decline in transketolase activity resulting from thiamine deficiency has even been found in various brain areas of alcoholics who do not exhibit the clinical and neuropathological signs of WE (Lavoie and Butterworth 1995), suggesting that thiamine deficiency can cause adverse effects even before severe brain damage becomes obvious.

### Thiamine Uptake Into the Cell

Thiamine is ingested with the diet, and to exert its effects in the cells it must be transported from the gastrointestinal tract to the tissues and cells. This transport involves at least four steps:

- Uptake from the intestine into the cells that line the intestine
- Transport out of those cells into the bloodstream
- Uptake from the blood into the tissues and cells; for thiamine transported to the brain this also includes crossing the blood–brain barrier
- Transport within the cells to the areas where the thiamine is needed (e.g., to the cell’s energy factories, the mitochondria, where PDH and α-KGDH act, or to the nucleus, where thiamine regulates gene activity).

These transport steps are accomplished by one or more thiamine transporter molecules. Researchers recently have identified and cloned the gene for a human thiamine transporter (see Singleton and Martin 2001). However, the characteristics of the thiamine transport process differ among different tissues and cell types, suggesting that variants of one transporter type or even different types of transporters may exist. Indeed, a second thiamine transporter gene recently has been cloned (Rajgopal et al. 2001). As will be described in more detail in the section “Differential Sensitivity to Thiamine Deficiency,” subtle variations in the transporter molecule among cells or among people, resulting in a reduced capacity to transport thiamine, may contribute to the differential sensitivity to thiamine deficiency.

Once taken up into the cells, thiamine first is modified by the addition of one or more phosphate groups. The compound containing two phosphate groups (thiamine diphosphate [ThDP]) is the actual active molecule that serves as a cofactor for the various thiamine-requiring enzymes. The levels of phosphate-free thiamine in the cell are relatively low and are tightly regulated by rapid conversion to the phosphorylated forms.

### Mechanisms of Thiamine Deficiency–Induced Cell Damage

Thiamine deficiency can lead to cell damage in the central nervous system through several mechanisms. First, the changes in carbohydrate metabolism, particularly the reduction in α-KGDH activity, can lead to damage to the mitochondria. Because the mitochondria produce by far the most energy required for cellular function, mitochondrial damage can result in cell death through a mechanism called necrosis (see Singleton and Martin 2001). Second, disturbances associated with thiamine deficiency in some cell types lead to apoptosis—a form of programmed cell death (or cell suicide) that serves to remove damaged cells from the organism (see Singleton and Martin 2001). Third, altered carbohydrate metabolism can lead to a cellular state called oxidative stress (Calingasan et al. 1999; Todd and Butterworth 1999), characterized by excess levels of highly reactive molecules called free radicals and/or the presence of insufficient levels of compounds to eliminate those free radicals (i.e., antioxidants, such as glutathione). Oxidative stress can lead to various types of cell damage and even cell death.

## Alcohol’s Effects on Thiamine Uptake and Function

As noted earlier, thiamine deficiency in affluent countries clearly is linked to alcoholism, occurring in up to 80 percent of alcoholics (e.g., Morgan 1982). However, only a subset of these alcoholics develop brain disorders such as WKS. Moreover, identical twins (who share all of their genetic information) show greater similarity with respect to alcohol-induced brain disease than do fraternal twins (who share on average 50 percent of their genetic information). These two observations have led to the conclusion that a genetic predisposition to thiamine deficiency and its effects may exist, as will be discussed in more detail in the section “Differential Sensitivity to Thiamine Deficiency.”

Research over the past 30 years has identified several mechanisms through which alcoholism may contribute to thiamine deficiency. The most important of these mechanisms (as discussed in Hoyumpa 1980) include:

- Inadequate nutritional intake
- Decreased absorption of thiamine from the gastrointestinal tract and reduced uptake into cells
- Impaired utilization of thiamine in the cells.

### Inadequate Nutritional Intake

Although most people require a minimum of 0.33 mg thiamine for each 1,000 kcal of energy they consume, alcoholics tend to consume less than 0.29 mg/1,000 kcal (Woodhill and Nobile 1972). In fact, in an early study of 3,000 alcoholics admitted to hospitals because of alcohol withdrawal symptoms or other alcohol-related illnesses, 40 percent exhibited periodic thiamine deficiency during drinking binges, 25 percent exhibited prolonged thiamine deficiency with some periods of normal intake, and 35 percent exhibited continuous thiamine deficiency (Leevy and Baker 1968). A later study found that alcoholic patients had significantly lower average levels of a thiamine compound containing one phosphate group (i.e., thiamine monophosphate), but the average levels of free thiamine and ThDP were similar in alcoholics and control subjects (Tallaksen et al. 1992). However, some of the alcoholics in that study had extremely high levels of free thiamine, suggesting that they may have had a problem in the steps that lead to the conversion of thiamine into its active, phosphate-containing form.

### Decreased Uptake of Thiamine From the Gastrointestinal Tract

Animal studies have helped elucidate the mechanisms of normal and alcohol-impaired thiamine uptake from the gastrointestinal tract into the blood and cells. To be used by the body, thiamine must cross a number of barriers, first transferring across the membranes of the cells lining the gut (i.e., enterocytes), then entering those cells, and then crossing the membranes at the other end of the cells to enter the bloodstream. At low thiamine concentrations, such as those normally found in the human body, this transfer is achieved by a specific thiamine transporter molecule that requires energy. This is called an active transport process and seems to be associated with the rapid addition of two phosphate groups by the enzyme thiamine diphosphokinase (TPK) once the thiamine is inside the cell. At high thiamine concentrations, however, such as can be achieved after additional thiamine is administered, thiamine transport occurs through a passive process—that is, a mechanism that requires no energy.

Acute alcohol exposure interferes with the absorption of thiamine from the gastrointestinal tract at low, but not at high, thiamine concentrations (Hoyumpa 1980). Furthermore, in studies using rats, the activity of the TPK enzyme from various tissues decreased with acute alcohol exposure to about 70 percent of the activity level in control animals, and with chronic alcohol exposure to about 50 percent (Laforenza et al. 1990). Although no studies have addressed whether alcohol directly affects TPK in humans, indirect analyses have found that the ratio of phosphorylated thiamine (primarily ThDP) to thiamine is significantly lower in alcoholics than in nonalcoholics (Poupon et al. 1990; Tallaksen et al. 1992)—that is, that less thiamine is converted to ThDP. This finding suggests that TPK is less active in the alcoholics.

Thiamine malabsorption could become clinically significant if combined with the reduced dietary thiamine intake that is typically found in alcoholics, when other aspects of thiamine utilization are compromised by alcohol, or when a person requires increased thiamine amounts because of his or her specific metabolism or condition (e.g., in pregnant or lactating women).

### Impaired Thiamine Utilization

The cells’ utilization of thiamine can be affected in different ways by chronic alcohol use. As mentioned earlier, once thiamine is imported into the cells, it is first converted into ThDP by the addition of two phosphate groups. ThDP then binds to the thiamine-using enzymes, a reaction that requires the presence of magnesium. Chronic alcohol consumption frequently leads to magnesium deficiency, however (Morgan 1982; Rindi et al. 1992), which also may contribute to an inadequate functioning of the thiamine-using enzymes and may cause symptoms resembling those of thiamine deficiency. In this case, any thiamine that reaches the cells cannot be used effectively, exacerbating any concurrently existing thiamine deficiency.

Abstinence from alcohol and improved nutrition have been shown to reverse some of the impairments associated with thiamine deficiency, including improving brain functioning (Martin et al. 1986). Researchers also administered thiamine to alcoholic patients and laboratory animals and found that this treatment reversed some of the behavioral and metabolic consequences of thiamine deficiency (Victor et al. 1989; Lee et al. 1995). Most recently, researchers administered different thiamine doses for two days to a group of alcoholics undergoing detoxification, none of whom were diagnosed with WKS, and then tested the participant’s working memory. These studies found that participants who received the highest thiamine dose performed best on tests of working memory (Ambrose et al. 2001).

## Differential Sensitivity to Thiamine Deficiency

### Differences in Sensitivity Among People

Several findings suggest that not all people are equally sensitive to thiamine deficiency and its consequences. For example, although thiamine deficiency may occur in up to 80 percent of alcoholics (Tallaksen et al. 1992; Hoyumpa 1980; Morgan 1982), only about 13 percent of alcoholics develop WKS (Harper et al. 1988). This means that the severest consequences of thiamine deficiency develop only in a subset of people who consume alcohol and have poor nutrition on a chronic basis. A possible explanation for this differential sensitivity is that some people are genetically predisposed to develop brain damage after experiencing repeated episodes of alcohol-related thiamine deficiency. To investigate this hypothesis, researchers have studied the activities of thiamine-using enzymes in patients with and without Korsakoff ’s psychosis, arguing that variants of these enzymes may exist that could differ in their susceptibility to thiamine deficiency. The results of these investigations, however, have been inconsistent.^2^

One study (Blass and Gibson 1977) compared the activity of transketolase, PDH, and α-KGDH derived from skin cells of people with and without Korsakoff ’s psychosis. These investigators found that transketolase from the Korsakoff ’s patients bound ThDP less avidly than did the enzyme from the control subjects. Transketolase from the Korsakoff ’s patients could function normally when sufficient thiamine or ThDP was present; under conditions of thiamine deficiency, however, the transketolase molecules would not be able to bind enough ThDP to maintain normal enzyme activity. As a result, the Korsakoff ’s patients would be more susceptible to developing complications of thiamine deficiency than would people with a transketolase variant that more readily binds ThDP. The investigators found no differences, however, between Korsakoff ’s patients and control subjects in the ability of the PDH and α-KGDH enzymes to bind ThDP.

In another study (Mukherjee et al. 1987), researchers studied transketolase activity in alcoholic men without Korsakoff ’s psychosis and their sons who had not yet been exposed to alcohol (i.e., who were alcohol naive) and compared it with transketolase activity in nonalcoholic volunteers and their sons. This analysis found that the enzyme from the alcoholic men and their sons also bound ThDP less strongly than did the enzyme from the healthy volunteers and their sons (fathers and sons were similar to each other in both groups). This finding suggests that the genetic makeup of alcoholics or those who are at risk of becoming alcoholic (e.g., sons of alcoholics who are still alcohol naive) might cause them to be more affected by thiamine deficiency than nonalcoholics.

Other investigators, however, have found no differences in the ability of transketolase from Korsakoff ’s patients and healthy subjects to bind ThDP (Nixon et al. 1984). Several reasons may explain these differences in findings. For example, if a study includes active alcoholics, toxic substances formed during alcohol degradation in the body (e.g., acetaldehyde or oxygen radicals) could conceivably damage the transketolase, leading to impaired transketolase activity even if the person does not have a genetic predisposition. Moreover, processing of the samples being studied could have modified and deactivated the transketolase. Overall, researchers to date have found no consistent correlation between genetically determined transketolase variants and a person’s sensitivity to thiamine deficiency (McCool et al. 1993). To determine whether a genetic predisposition to thiamine deficiency and resulting brain damage does indeed exist, more detailed molecular genetic studies are required.

Another possible explanation for the differences among people in their sensitivity to thiamine deficiency has focused on the assembly of functional transketolase. To yield a functional enzyme, two transketolase molecules—each of which is bound to ThDP and to magnesium—must come together. This assembly step is aided by an as yet unidentified “assembly factor,” which is probably also involved in the assembly of other thiamine-using enzymes. If this factor were defective, the final enzyme complex would be formed at a lower rate and would be unstable (Wang et al. 1997). Researchers have identified at least one person with WKS whose cells showed enhanced sensitivity to thiamine deficiency and in whom the assembly factor was defective (Wang et al. 1997). Other mechanisms that could contribute to individual differences in the sensitivity to alcoholism could involve variability in the capacity for thiamine uptake into the cells or in the overall sensitivity to cell damage induced by oxidative stress.

### Differential Sensitivity of Various Brain Regions

Various brain regions and even different cell types within one brain region may differ in their sensitivity to alcohol-induced damage as well as in their susceptibility to associated problems, including alcohol-related malnutrition (e.g., thiamine deficiency). For example, as mentioned earlier, the cerebellum appears to be particularly sensitive to thiamine deficiency, as indicated by the high frequency of cerebellar degeneration in alcoholics. Autopsy studies have found that a region of the cerebellum known as the anterior superior cerebellar vermis most frequently exhibits alcohol-induced damage (Baker et al. 1999). Additional studies have found that the cerebellar vermis is particularly sensitive to the deleterious effects of thiamine deficiency (Baker et al. 1999; Lavoie and Butterworth 1995; Victor et al. 1989). For example, thiamine deficiency contributes to a reduction in the number and size of a certain cerebellar cell type called Purkinje cells in parts of the cerebellar vermis (Philips et al. 1987).

The sensitivity of the cerebellum to alcohol-related damage was confirmed in a recent study in which investigators used an imaging technique called proton magnetic resonance spectroscopy (proton MRS) to determine the levels of certain molecules (i.e., metabolites) that reflect the functionality of the cells in various brain regions of alcoholics and nonalcoholics. For example, one metabolite reflects nerve cell activity, another metabolite reflects the degradation and formation (i.e., turnover) of cell membrane components, and a third metabolite reflects cellular energy levels. The results of the analyses indicated that these metabolites are significantly reduced in the cerebellum of alcoholics, more so than in another brain region commonly affected by alcohol, the frontal white-matter cortex (Parks et al. 2002). Moreover, only some of these reductions in metabolite levels were reversed when the subjects were tested again after 3 weeks and then 3 months of abstinence. These findings suggest that the cerebellum, in particular the cerebellar vermis, is uniquely sensitive to alcohol’s effects, including alcohol-related thiamine deficiency, and therefore may be the initial target of alcohol-related damage.

This hypothesis is consistent with the clinical course of the neurocognitive deficits observed in alcoholics. Networks of nerve cells (i.e., neural pathways) extend from the cerebellum through brain regions called the basal ganglia and thalamus to the frontal lobe. These pathways mediate not only traditional cerebellar functions, such as motor control, but also perceptual–motor tasks, executive functions, and learning and memory, all of which are impaired in alcoholics (see Parks et al. 2002). Accordingly, alcohol-induced damage to the cerebellar vermis could indirectly affect neurocognitive functions attributed to the frontal lobe, even early in the disease process when no cortical damage is detectable, by disrupting the neural pathways connecting the two brain regions. As the alcoholism progresses and alcohol exposure persists, damage to the frontal lobe is also likely to occur, further interfering with the functions of that brain region.

In addition to the cerebellum, numerous other brain regions and structures are damaged in people with WKS. Although animal studies have suggested that thiamine deficiency may contribute to damage to these structures, the exact role of thiamine deficiency and the level of sensitivity of these structures to thiamine deficiency have not yet been determined. Further studies are certainly needed in this area.

## Summary

Thiamine deficiency, which is found in a large number of alcoholics, is an important contributor to alcohol-related brain damage of all kinds, not only WKS, as was commonly thought in the past. Thiamine is an essential cofactor for several enzymes involved in brain cell metabolism that are required for the production of precursors for several important cell components as well as for the generation of the energy-supplying molecule ATP. Thiamine deficiency leads to significant reductions in the activities of these enzymes, and to deleterious effects on the viability of brain cells.

Chronic alcohol consumption can cause thiamine deficiency and thus reduced enzyme activity through several mechanisms, including inadequate dietary intake, malabsorption of thiamine from the gastrointestinal tract, and impaired utilization of thiamine in the cells. Accordingly, thiamine deficiency can potentiate a number of processes associated with chronic alcohol consumption that are toxic to brain cells, as discussed in other articles in this journal issue. It is important to note that these adverse effects of alcohol-induced thiamine deficiency, particularly the reduction of transketolase activity, can occur even in alcoholics who do not show evidence of WE or WKS.

The extent to which alcohol exerts its detrimental effects on the brain and various other tissues may be genetically determined via individual differences in predisposition to thiamine deficiency disorders. For example, some studies have suggested that there may be different variants of the genes encoding transketolase, which differ in their ability to bind the active form of thiamine, particularly at low thiamine concentrations. Such a genetic variation could be one explanation for why only a subset of alcoholics who experience thiamine deficiency develop the pathological consequences of that condition, such as WKS. Additional genetic studies are necessary, however, to clarify the roles of different genetic variants and determine whether a genetically determined susceptibility does indeed exist.

Various brain regions also differ in their sensitivity to alcohol’s effects, including alcohol-induced thiamine deficiency. The cerebellum appears to be particularly sensitive to the effects of thiamine deficiency and is the region most frequently damaged in association with chronic alcohol consumption. This heightened susceptibility is consistent with the cognitive deficits typically associated with alcoholism. These deficits are indicative either of cerebellar damage or of damage to the frontal lobes, which are connected to the cerebellum through neural pathways. Accordingly, reversal of thiamine deficiency—for example, by administering thiamine at pharmacological levels— may not only ameliorate the consequences of cerebellar damage but improve some brain functions typically associated with the frontal lobe.

## Footnotes

1 Lower levels are recommended for children, and slightly higher levels (1.4 mg thiamine per day) are recommended for pregnant and breast-feeding women.

2 The studies cited in this section mostly used enzymes isolated from skin or blood cells of the participants. Although it is not known whether the effects of thiamine deficiency on these cells are identical to those on brain cells, the thiamine-using enzymes in these cells should be similar to the enzymes in brain cells, which are not accessible to the researchers. Using such model systems to investigate mechanisms of cell function has a long tradition in research.

## References

[b1-134-142] Ambrose ML, Bowden SC, Whelan G (2001). Thiamin treatment and working memory function of alcohol-dependent people: Preliminary findings. Alcoholism: Clinical and Experimental Research.

[b2-134-142] Baker KG, Harding AJ, Halliday GM (1999). Neuronal loss in functional zones of the cerebellum of chronic alcoholics with and without Wernicke’s encephalopathy. Neuroscience.

[b3-134-142] Blass JP, Gibson GE (1977). Abnormality of a thiamine-requiring enzyme in patients with Wernicke-Korsakoff syndrome. New England Journal of Medicine.

[b4-134-142] Calingasan NY, Chun WJ, Park LC (1999). Oxidative stress is associated with region-specific cell death during thiamine deficiency. Journal of Neuropathology and Experimental Neurology.

[b5-134-142] Charness ME (1993). Brain lesions in alcoholics. Alcoholism: Clinical and Experimental Research.

[b6-134-142] Ferguson RK, Soryal IN, Pentland B (1997). Thiamine deficiency in head injury: A missed insult?. Alcohol and Alcoholism.

[b7-134-142] Gibson GE, Ksiezak-Reading H, Sheu K-FR (1984). Correlation of enzymatic, metabolic, and behavioral deficits in thiamine deficiency and its reversal. Neurochemical Research.

[b8-134-142] Harper C (1998). The neuropathology of alcohol-specific brain damage, or does alcohol damage the brain?. Journal of Neuropathology and Experimental Neurology.

[b9-134-142] Harper C, Rodriguez M, Gold J, Perdices M (1988). The Wernicke-Korsakoff syndrome in Sydney—a prospective necropsy study. Medical Journal of Australia.

[b10-134-142] Hoyumpa AM (1980). Mechanisms of thiamine deficiency in chronic alcoholism. American Journal of Clinical Nutrition.

[b11-134-142] Hung SC, Hung SH, Tang DC (2001). Thiamine deficiency and unexplained encephalopathy in hemodialysis and peritoneal dialysis patients. American Journal of Kidney Disease.

[b12-134-142] Laforenza U, Patrini C, Gastaldi G, Rindi G (1990). Effects of acute and chronic ethanol administration on thiamine metabolizing enzymes in some brain areas and in other organs of the rat. Alcohol and Alcoholism.

[b13-134-142] Lavoie J, Butterworth RF (1995). Related activities of thiamine-dependent enzymes in brains of alcoholics in the absence of Wernicke’s encephalopathy. Alcoholism: Clinical and Experimental Research.

[b14-134-142] Lee H, Tarter J, Holburn G (1995). In vivo localized proton NMR spectroscopy of thiamine-deficient rat brain. Magnetic Resonance Medicine.

[b15-134-142] Leevy CM, Baker H (1968). Vitamins and alcoholism. American Journal of Clinical Nutrition.

[b16-134-142] Martin PR, Adinoff B, Weingartner H (1986). Alcoholic organic brain disease: Nosology and pathophysiologic mechanisms. Progress in Neuropsychopharmacological and Biological Psychiatry.

[b17-134-142] McCool SG, Plonk SG, Martin PR, Singleton CK (1993). Cloning of human transketolase cDNAs and comparison of the nucleotide sequence of the coding region in Wernicke-Korsakoff and non-Wernicke-Korsakoff individuals. Journal of Biological Chemistry.

[b18-134-142] Morgan MY (1982). Alcohol and nutrition. British Medical Bulletins.

[b19-134-142] Mukherjee AB, Svoronos S, Ghazanfari A (1987). Transketolase abnormality in cultured fibroblasts from familial chronic alcoholic men and their male offspring. Journal of Clinical Investigation.

[b20-134-142] Nixon PF, Kaczmarek MJ, Tate J (1984). An erythrocyte transketolase isoenzyme pattern associated with the Wernicke-Korsakoff syndrome. European Journal of Clinical Investigation.

[b21-134-142] Parks MH, Dawant BM, Riddle WR (2002). Longitudinal brain metabolic characterization of chronic alcoholics with proton magnetic resonance spectroscopy. Alcoholism: Clinical and Experimental Research.

[b22-134-142] Parks MH, Morgan VL, Pickens DR (2003). Brain FMRI activation associated with self-paced finger tapping in chronic alcohol-dependent patients. Alcoholism: Clinical and Experimental Research.

[b23-134-142] Philips SC, Harper C, Kril J (1987). A quantitative histological study of the cerebellar vermis in alcoholic patients. Brain.

[b24-134-142] Poupon RE, Gervaise G, Riant P (1990). Blood thiamine and thiamine phosphate concentrations in excessive drinkers with or without peripheral neuropathy. Alcohol and Alcoholism.

[b25-134-142] Ragan PW, Singleton CK, Martin PR (1999). Brain injury associated with chronic alcoholism. CNS Spectrums.

[b26-134-142] Rajgopal A, Edmondson A, Goldman ID, Zhao R (2001). SLC19A3 encodes a second thiamine transporter ThTr2. Biochimica et Biophysica Acta.

[b27-134-142] Rindi G, Casirola D, Poggi V (1992). Thiamine transport by erythrocytes and ghosts in thiamine-responsive megaloblastic anemia. Journal of Inherited Metabolic Diseases.

[b28-134-142] Singleton CK, Martin PR (2001). Molecular mechanisms of thiamine utilization. Current Molecular Medicine.

[b29-134-142] Tallaksen CME, Bohmer T, Bell H (1992). Blood and serum thiamin and thiamin phosphate esters concentrations in patients with alcohol dependence syndrome before and after thiamin treatment. Alcoholism: Clinical and Experimental Research.

[b30-134-142] Todd K, Butterworth R (1999). Early microglial response in experimental thiamine deficiency: An immunohistochemical analysis. Glia.

[b31-134-142] Torvik A (1987). Brain lesions in alcoholics: Neuropathological observations. Acta Medica Scandinavica.

[b32-134-142] Victor M, Davis RD, Collins GH (1989). The Wernicke-Korsakoff Syndrome and Related Neurologic Disorders Due to Alcoholism and Malnutrition.

[b33-134-142] Wang JJ-L, Martin PR, Singleton CK (1997). A transketolase assembly defect in a Wernicke-Korsakoff syndrome patient. Alcoholism: Clinical and Experimental Research.

[b34-134-142] Woodhill JM, Nobile S (1972). Thiamine in the 1970 Australian diet with special reference to cereals and the assessment of thiamine status. International Journal of Vitamin and Nutrition Research.

